# Co-expression analysis suggests lncRNA-mRNA interactions enhance antiviral immune response during acute Chikungunya fever in whole blood of pediatric patients

**DOI:** 10.1371/journal.pone.0294035

**Published:** 2023-11-03

**Authors:** Juliana de Souza Felix, Mariana Cordeiro Almeida, Maria Fernanda da Silva Lopes, Flávia Regina Florencio de Athayde, Jéssica Antonini Troiano, Natália Francisco Scaramele, Amanda de Oliveira Furlan, Flavia Lombardi Lopes

**Affiliations:** School of Veterinary Medicine, Araçatuba, Department of Production and Animal Health, São Paulo State University (Unesp), São Paulo, Brazil; University of Sri Jayewardenepura, SRI LANKA

## Abstract

Chikungunya virus is an arbovirus that causes the neglected tropical disease chikungunya fever, common in tropical areas worldwide. There is evidence that arboviruses alter host transcriptome and modulate immune response; this modulation may involve transcriptional and post-transcriptional control mechanisms mediated by long non-coding RNAs (lncRNAs). Herein, we employed bioinformatic analysis to evaluate co-expression of lncRNAs and their putative target mRNAs in whole blood during natural Chikungunya infection in adolescent boys. Sequencing data from GSE99992 was uploaded to the Galaxy web server, where data was aligned with HISAT2, gene counts were estimated with HTSeq-count, and differential expression was run with DESeq2. After gene classification with Biomart, Pearson’s correlation was applied to identify potential interactions between lncRNAs and mRNAs, which were later classified into cis and trans according to genomic location (FEELnc) and binding potential (LncTar), respectively. We identified 1,975 mRNAs and 793 lncRNAs that were differentially expressed between the acute and convalescent stages of infection in the blood. Of the co-expressed lncRNAs and mRNAs, 357 potentially interact in trans and 9 in cis; their target mRNAs enriched pathways related to immune response and viral infections. Out of 52 enriched KEGG pathways, the RIG-I like receptor signaling is enriched by the highest number of target mRNAs. This pathway starts with the recognition of viral pathogens, leading to innate immune response mediated by the production of IFN-I and inflammatory cytokines. Our findings indicate that alterations in lncRNA expression in adolescent boys, induced by acute Chikungunya infection, potentially modulate mRNAs that contribute to antiviral immune responses.

## Introduction

The reemerging mosquito-borne alphavirus known as the Chikungunya virus (CHIKV) is responsible for epidemics affecting tropical areas of the world. CHIKV is mainly spread by infected mosquitoes belonging to the *Aedes* species (*A*. *aegypti* and *A*. *albopictus*) [1]. According to the European Center for Disease Prevention and Control (2022) [2], 113,052 Chikungunya cases were reported in 15 countries up to June 2022, with the vast majority of cases (92,349), as well as the 14 reported deaths coming from Brazil.

Most CHIKV infections are symptomatic, and common clinical manifestations are high fever, rash, headache, and severe polyarthralgia that can last from months to years [3]. Arthralgia lasting longer than three months characterizes the chronic stage of Chikungunya. Acute infection might last up to 21 days, and viremia is prevalent for 5–7 days, although intensity of symptoms usually decreases after a couple of weeks [4]. Currently, there are no vaccines or specific drugs against CHIKV approved for use in humans, and treatment is based on symptom relief.

Understanding the dynamics of host-pathogen interaction can help the search for potential therapeutic targets. Several reports evidence that viral infections significantly alter host transcriptome, including CHIKV [5–7]. These changes may be related to transcriptional and post-transcriptional control mechanisms mediated by long non-coding RNAs (lncRNAs) in several ways [8, 9]. LncRNAs are tissue-specific transcripts with poor coding potential, and can be classified as *cis* or *trans* based on the chromosomal distance to their targets. *Cis*-acting lncRNAs are those that act at the same loci where they are transcribed [10]; in turn, *trans*-acting lncRNAs are transcribed, processed, and then leave their transcription site to execute their function in another region, either in the nucleus or in the cytoplasm of the cell [11]. Several lncRNAs were identified in response to infection by other arboviruses, including Dengue and Zika Virus, indicating that altered lncRNA expression may participate in host response and immunity [12, 13].

In view of the role of lncRNAs in epigenetic regulation during other arboviral infections, we aimed to elucidate whether the expression of lncRNAs is correlated with presumptive target mRNAs during natural Chikungunya infection in pediatric patients and whether these interactions may play key roles in the pathogenesis of the disease and antiviral response.

Here, we present lncRNA expression profiles in whole blood during acute and convalescent Chikungunya infection in adolescent boys. We identify co-expressed lncRNAs-mRNAs in *cis* and *trans* regulation, enriching viral and immune responses related pathways.

## Methods

### Dataset

Publicly available RNA-Seq data were collected from the Gene Expression Omnibus (GEO) database, bioproject PRJNA390289 and accession number GSE99992. To generate this data, Michlmayr et al. (2018) [7] performed whole-blood RNA-Seq of acute and convalescent phase samples from 42 spontaneously infected children with CHIKV. Acute phase samples were taken on days 1–2 post-symptom onset, and convalescent phase samples of these same individuals were taken on days 15–17, after the resolution of symptoms and viremia. To decrease confounding factors, we did not use samples without acute viral load and convalescent IgG titer and samples from children under 11 years of age. Samples from girls were removed due to hormonal variations, which are known to influence epigenetic mechanisms. A total of 34 samples (17 from each phase) collected from adolescent boys (11–15 years old) were used for our analyses.

### RNA-seq analysis

Paired-end FASTQ files from both phases of illness were extracted from the sequence read archive (SRA) database using Download and Extract Reads in the FASTA/Q (Galaxy Version 2.10.8+galaxy0) tool, available at the Galaxy web platform (www.usegalaxy.org) [14]. Quality of reads was checked with FastQC (Galaxy Version 0.73+galaxy0), reads with Phred score > 28 and average read length of 140bp were considered for further analysis. Reads were then aligned to the human genome reference (assembly GENCODE GRCh38.p13) using HISAT2 (Galaxy Version 2.1.0+galaxy5) [15]. Read counts were performed using HTSeq-count (Galaxy Version 0.9.1) with comprehensive gene annotation GENCODE V34 [16]. Quality reports of all steps were analyzed with MultiQC (Galaxy Version 1.8+galaxy1) [17].

### Differential gene expression analysis

Using DESeq2 (Galaxy Version 2.11.40.6+galaxy1), we estimated mean-variance dependency in count data and used the Wald test to identify transcripts that were differentially expressed (FDR<0.05; log2(FC)≥1) between acute and convalescent phases of the disease [18]. Next, we used the BioMart tool (release 103) to obtain the classification of our differentially expressed transcripts according to their biotype [19]. Annotation relied on Ensembl coding/non-coding classification, and those transcripts of the “lncRNA” type were considered as lncRNAs, while those of the “protein_coding” type were considered as mRNAs.

### Co-expression analysis of lncRNAs and mRNAs

Due to the scarcity of comprehensive databases that serve as a resource for experimentally verified lncRNA function, co-expression analysis is often employed to infer the role of lncRNAs in all biological and pathological contexts. To identify co-expressed lncRNA-mRNA pairs, Pearson correlation coefficients were calculated based on the expression value of every differentially expressed (DE) lncRNA and mRNA pair. Threshold for Pearson correlation coefficient was set to |r| ≥ 0.95 and the corresponding FDR was set to <0.01. Using the FEELnc (Flexible Extraction of Long non-coding RNAs) tool, the putative interactions between lncRNA-mRNA were classified as *cis*- or *trans*-acting, based on the chromosomal distance between the transcripts, with the FEELnc classifier module, considering a sliding window of up to 100kbps in distance [20]. Further, potential of ligation between *trans* lncRNAs and putative target mRNAs with significant correlation was evaluated based on the estimates of free energy (normalized dG (ndG) was set to -0.10), using the LncTar tool [21].

### Functional enrichment analysis

All mRNAs targeted by lncRNAs for which the criteria of co-expression (FDR<0.01 and |r|≥0.95) and potential of ligation (for all *trans*-acting transcripts), or nearby genes (for all *cis*-acting transcripts) were met, were analyzed by Kyoto Encyclopedia of Genes and Genomes (KEGG), REACTOME, and Gene Ontology (GO) using the g:GOSt tool in g:Profiler web server, applying the Benjamini-Hochberg FDR multiple testing correction method set at 0.05 [22].

### Construction of lncRNA-mRNA co-expression network

Gene co-expression network were built using the Cytoscape software (version 3.9.0), and the top five KEGG pathways, according to the highest percentage of our target mRNAs in the pathways, were evaluated. Cytoscape combines all the identified interactions among the genes and generates the final integrated regulatory network.

**Fig 1:**
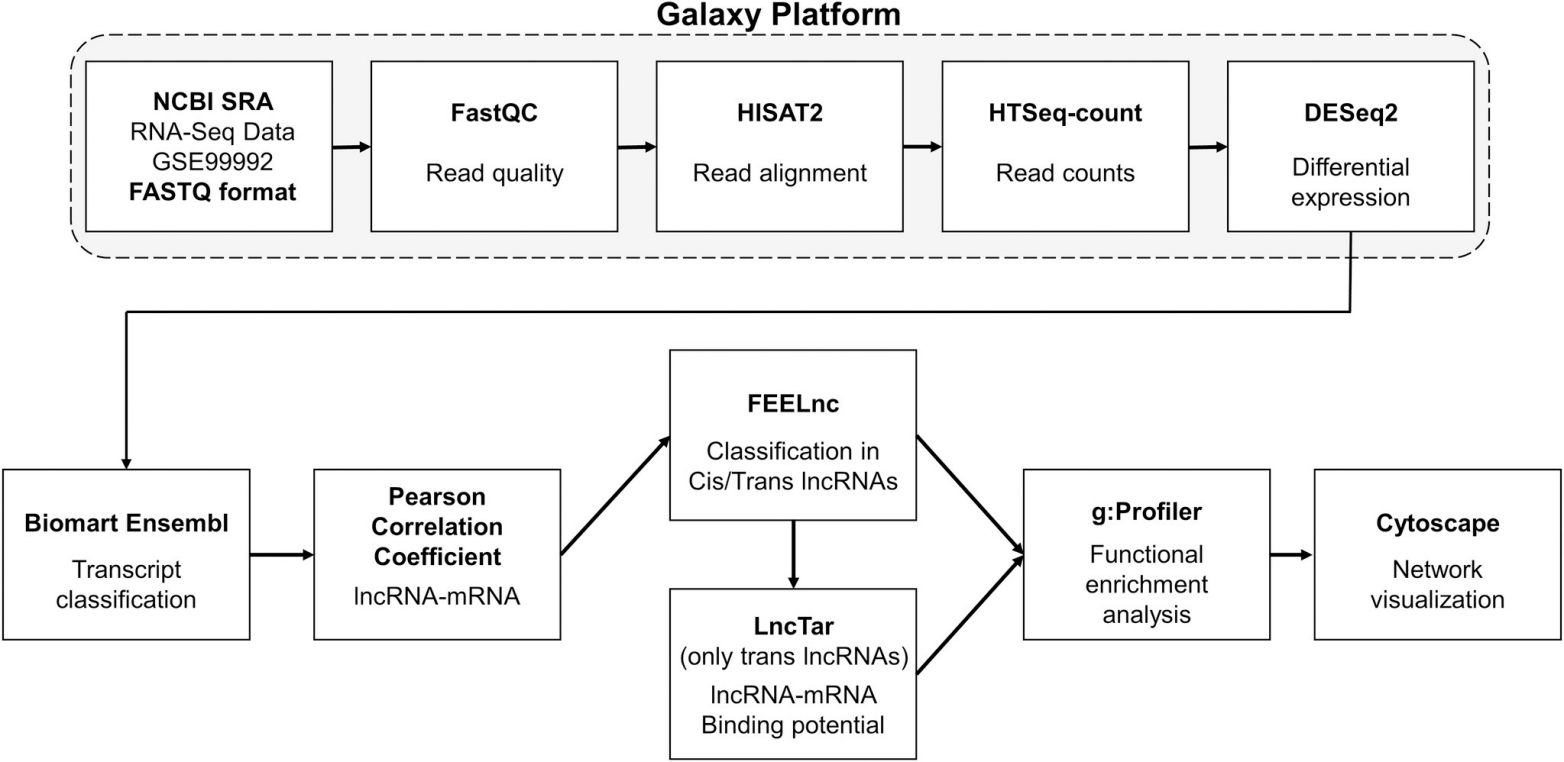
Workflow for lncRNA-mRNA co-expression analysis. All tools employed are summarized in this pipeline: read quality, alignment and counts, differential expression analysis, transcript classification, correlation, binding potential of lncRNAs, identification of lncRNAs *cis* targets, and enrichment analysis.

All bioinformatics tools used for the analysis, from read alignment to pathways, are depicted in a workflow in Fig 1.

## Results

**Fig 2:**
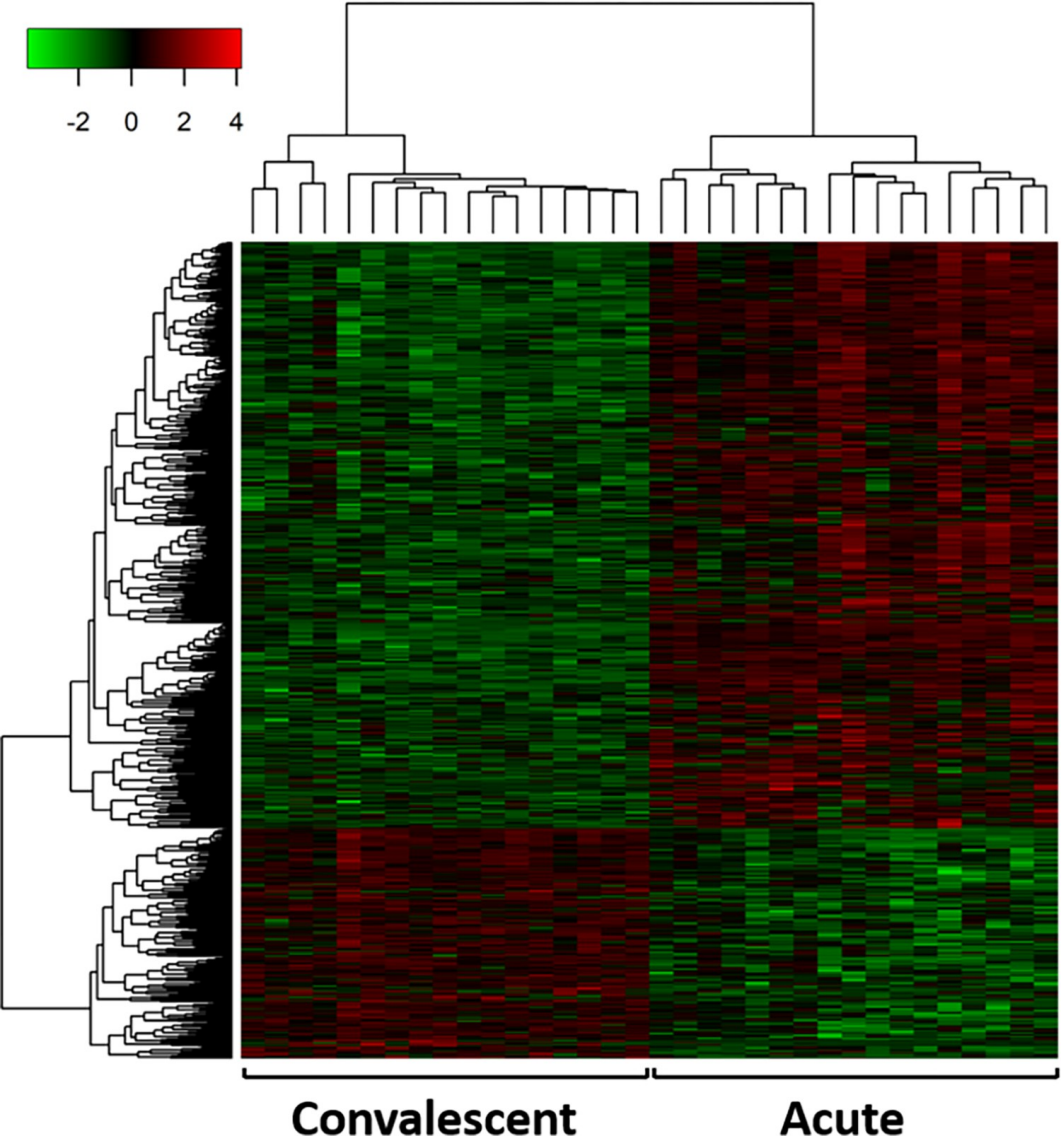
Heatmap of differentially expressed lncRNAs between acute and convalescent phases of chikungunya infection. Expression of 793 lncRNAs, DE in whole blood of male children aged 11 to 15 years old, is hierarchically grouped (hierarchical clustering used the complete linkage method with Euclidean distance) and represented according to the color scale in z-score (green indicates lower expression, whereas red indicates higher expression).

Out of all 3,461 differentially expressed transcripts (FDR<0.05; log2(FC)≥1) in whole blood of adolescent boys positive for CHIKV between acute and convalescent stages, 1,975 transcripts were classified as mRNAs (S1 Table) and 793 as lncRNAs (S2 Table), according to the Ensembl definition, available in the BioMart tool. Regarding those 793 lncRNAs, 569 DE lncRNAs showed elevated expression, while 224 were downregulated in the acute phase (Fig 2).

From a correlation matrix containing 774 potential lncRNA-mRNA interactions (|r|≥0.95; FDR<0.01) (S3 Table), we classified the distance between lncRNA and the putative mRNA based on the FEELnc algorithm, which resulted in 9 lncRNAs-mRNAs interactions predicted to have *cis* activity (S4 Table). We performed free energy analysis in LncTar (ndG≤-0.10), which resulted in 359 interactions with a potential of ligation between lncRNA-mRNA (S5 Table). Of these, 357 interactions occurred between genes outside the 100 kbps window and were considered to have *trans* activity.

**Fig 3:**
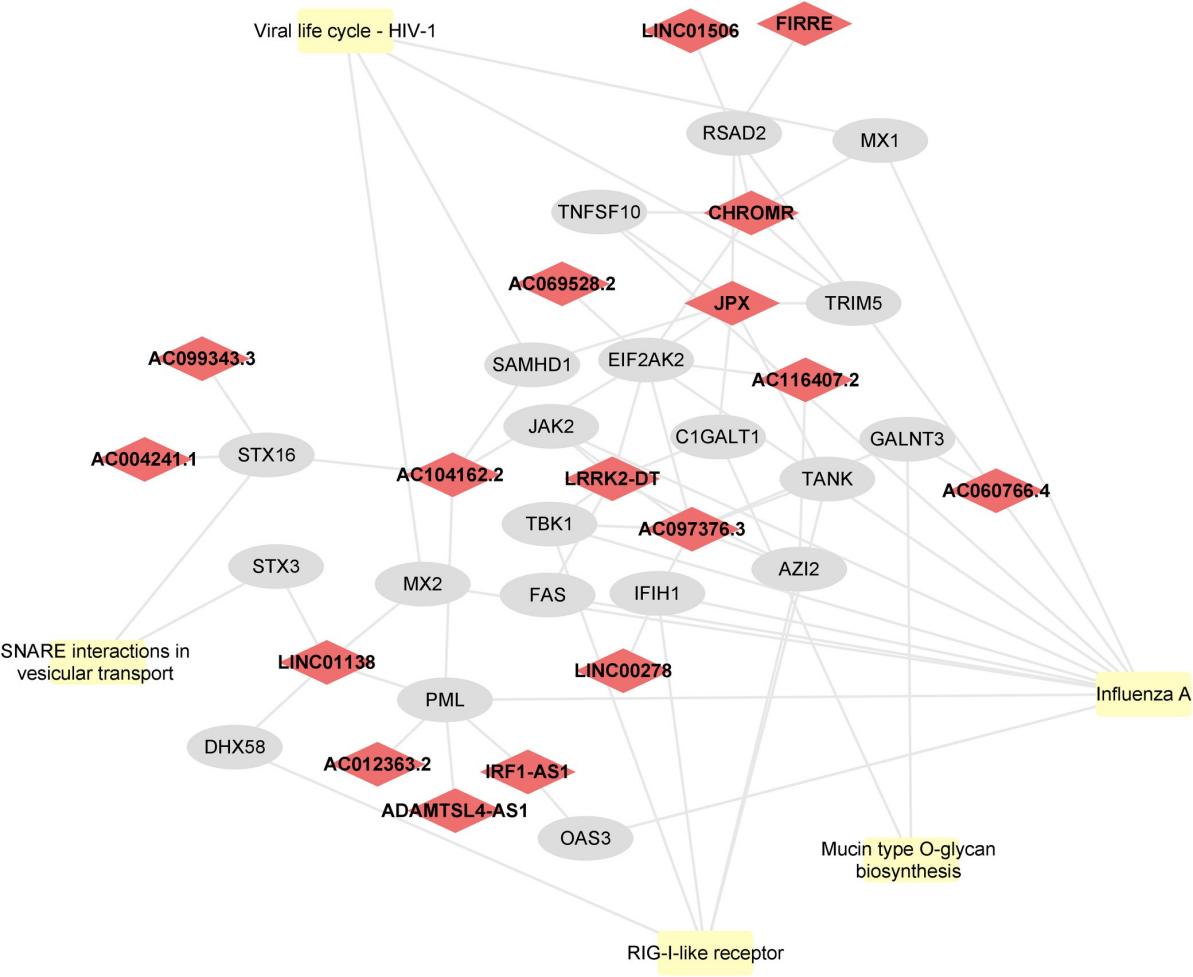
Network analysis of the top 5 pathways and related mRNAs and lncRNAs. All lncRNAs and mRNAs depicted were upregulated in acute phase of CHIKV infection. Node shape legends: Ellipses correspond to mRNAs; Diamonds to lncRNAs; Round rectangles to KEGG pathways.

After pathway enrichment analysis, we identified 266 REACTOME pathways, primarily related to immune response, including: Interferon Signaling, Interferon alpha/beta signaling, and Antiviral mechanism by IFN-stimulated genes (S6A Table). Also, 277 GO terms of molecular function were enriched, 1579 GO of biological processes, and 186 GO of cellular component (S6B Table). Moreover, 52 KEGG pathways were significantly enriched by 78 DE mRNAs, which were potentially regulated by 43 DE lncRNAs (S7 Table). These pathways are mainly related to immune response and viral infections. To better visualize the relationship between co-expressed transcripts, Cytoscape was used to assemble the top five KEGG pathways (Fig 3) in a network, ranked according to the highest ratio obtained using significant target mRNAs over the total number of genes in the pathway.

Given the relevance of the type I interferon response to viral infections, we used the INTERFEROME database v2.01 (INTERFEROME.org) to determine which of our lncRNAs and mRNAs are classified as ISGs [23]. The criteria for inclusion was any experiment where IFN (type I, II, or III) was employed and genes were DE on human blood cells. Among our 366 significant interactions between lncRNA-mRNA (9 *cis* and 357 *trans* interactions), we identified 160 ISGs, 154 were classified as mRNAs and 6 as lncRNA (S8 Table). These 6 ISGs lncRNAs are shown in Table 1. Furthermore, we performed an extensive literature search on all *cis* and *trans* DE lncRNAs and found that the lncRNA *BST2 IFN-Stimulated Positive Regulator* (*BISPR*) is also an ISG, although not yet included in the INTERFEROME database. The lncRNA *BISPR* interacts in *cis* with its neighboring gene *Bone Marrow Stromal Antigen 2* (*BST2*) (r = 0.90; p-value = 0.003) and interacts in *trans* with *2’-5’-Oligoadenylate Synthetase Like* (*OASL*) (r = 0.97; FDR = 2.03E-05).

**Table 1:** LncRNAs classified as ISGs according to INTERFEROME database.

lncRNA	*Trans* Target mRNAs
*CHROMR*	*LGALS8; CMPK2; RSAD2; EIF2AK2; PNPT1; NMI; DTX3L; HERC6; CD2AP; TENT5A; NCOA7; NT5C3A; PARP12; CHMP5; TMEM123; PARP11; SPATA13; ELF1; PHF11; STOML1; ADCY7; MT2A; MX1; KDM6A; PARP9; EPSTI1; ZNF200; BLZF1; TNFSF10; DAPP1; GLRX; HINT3; NUB1; TRIM5; XAF1; PANK2; ACOT9; CXorf21; SAMD9L; LMO2; SP100; FIG4; GBP4; IFIT3*
*LRRK2-DT*	*AZI2; NT5C3A; VMP1; MIER1; MCL1; LGALS8; CMPK2; EIF2AK2; HNRNPLL; RAB1A; RALB; NMI; ITPRID2; ATG3; PLSCR1; RNF13; GNB4; FAM241A; C4orf3; RAB33B; DDX60L; FAM8A1; CD2AP; CD164; C1GALT1; NAMPT; JAK2; CHMP5; FAS; EXOC6; PIK3AP1; TMEM123; TBK1; CDK17; SPATA13; PHF11; TNFSF13B; BAZ1A; TMEM62; ZNF267; RHOT1; H3-3B; SMCHD1; USP25; ACSL4; FMR1*
*IRF1-AS1*	*OAS3; PML; N4BP1; ANKFY1; AFF1*
*HCP5*	*SLC3A2; SASH3; CTSZ*
*CYTOR*	*TRAFD1; DOCK8; DENND5A; SBF2*
*ZFY-AS1*	*HESX1; MYL12A*

Next, we investigated *BISPR*/*OASL* and *BISPR*/*BST2* co-expression in other arboviral infections. Mainly transmitted by the *Aedes* species, Dengue fever (DENV) and Zika (ZIKV) infections present similar symptoms to Chikungunya infection and also lack specific treatment [24]. Public datasets using whole blood, and also comparing convalescent x acute disease stages, were analyzed using the GEO2R tool, available in the GEO database (significance level was set to FDR≤0.05) (S9 Table). Expression of *BISPR*, *OASL*, and *BST2* were upregulated during acute Dengue and Zika infection (Table 2), similarly to what we observed in acute Chikungunya infection.

**Table 2:** Expression of *BISPR*, *BST2*, and *OASL* in other arboviral diseases.

GEO Acession Number	Acute x Convalescent	*BISPR* Expression	*OASL* Expression	*BST2* Expression
GSE140809	Dengue	Log2FC = 1.314 p-adj = 2.55E-48	Log2FC = 2.349 p-adj = 1.38E-41	Log2FC = 1.549 p-adj = 2.28E-31
GSE129882	Zika	Log2FC = 1.582 p-adj = 1.07E-69	Log2FC = 3.154 p-adj = 3.42E-73	Log2FC = 1.817 p-adj = 2.51E-45

## Discussion

Previous reports revealed that numerous viral infections modify the host transcriptome, including both coding and non-coding transcripts [5–8]; moreover, lncRNAs induced by infection may influence viral pathogenesis and replication (as reviewed by Fortes and Morris (2016) [25]). Although intense research is being done on CHIKV infection and pathogenesis, the mechanisms mediating host-virus interactions are still elusive. Among several mechanisms involved in these interactions, altered expression of cellular lncRNA may follow CHIKV infection and can influence antiviral pathways induced by infection.

Therefore, using a publicly available RNA-Seq data produced by Michlmayr et al. (2018) [7], we analyzed the co-expression profiles of lncRNA-mRNA in whole blood obtained from adolescent boys naturally infected with CHIKV, in the acute and convalescent stages of the infection. Out of the 774 lncRNA-mRNA pairs, we identified 357 with potential for ligation, thus suggesting target regulation in *trans*, and 9 lncRNAs-mRNAs interactions in *cis*. Pathway enrichment analysis indicates that the co-expressed putative targets of the differentially expressed lncRNAs are involved in immune response and viral infection pathways mediated by ISGs.

RIG-I like receptor signaling pathway is enriched by five DE target mRNAs upregulated in acute stage of Chikungunya in whole blood (*AZI2*, *TBK1*, *TANK*, *IFIH1*, and *DHX58*). These five mRNAs were predicted in our analyzes to be *trans* targets of different DE lncRNAs. This pathway starts with viral pathogens recognition leading to innate immune response mediated by the production of IFN-I and inflammatory cytokines. *IFIH1* and *DHX58* are members of the retinoic acid-inducible gene-I-like receptors (RLRs) family, and are similar to the RIG-I receptor. *IFIH1* seems to play a similar regulatory role to RIG-I, each recognizing different types of viruses [26, 27]. The role of *DHX58* is not fully understood, but evidence suggests that it is complementary to *IFIH1*, and likely induces conformational changes in *IFIH1*, facilitating its interaction with viral RNA (reviewed by Thoresen et al. (2021) [28]). *TANK* e *AZI2* are involved in the activation of TBK1-mediated IFN regulatory factors (IRFs). TBK1 and IκB kinase-ε (IKKε) are NF-kB activators associated with phosphorylation of IRF3 and IRF7, triggering transcription of IFN-I (reviewed by Yoneyama and Fujita (2008) [29]). IFNs are notoriously important immune regulators of viral infections and appear to regulate *BISPR* expression [30, 31]. In the report that generated the dataset used for our analysis, Michlmayr et al. (2018) [7] found elevated serum levels of IFN-α and IFN-γ, which may be inducing the overexpression of *BISPR* reported in our study.

Our analysis reveals that *BISPR* was upregulated in the acute phase of CHIKV and is correlated in a *trans*-action with *OASL* (an ISG transcript), also upregulated in the acute phase. Viral infections lead to synthesis of IFN and IRF3, and these proteins lead to transcription of *OASL* [32]. By enhancing the sensitivity of Retinoic acid-inducible gene I (RIG-I) activation, OASL promotes antiviral host response. We suggest that the putative ligation between *BISPR* and *OASL* may support the stability of OASL in cytoplasm, increasing viral recognition by RIG-I and IFN synthesis.

OASL activates RIG-I by mimicking K63-linked polyubiquitin (pUb) to promote transcription of immunoregulatory antiviral genes. In the absence of viral nucleic acid, RIG-I is in a stable auto-inhibited conformation. The C-terminal domain of RIG-I detects and binds to viral RNA, in this case, causing a conformational shift in RIG-I, consequently exposing the RIG-I N-terminal caspase activation and recruitment domains (CARDs). CARDs then connect to pUb and convert RIG-I to an active state, enabling RIG-I to interact with a CARD domain also found in the mitochondrial antiviral-signaling protein, MAVS. MAVS are essential adaptor proteins for RIG-like receptors (RLR) signal transduction and, through activation of TBK1 and IKKε, activate IRF3 and IRF7, triggering transcription of *IFN-I*, *OASL*, and other antiviral immunoregulatory genes. After the onset of viral infection and stimulation of *OASL* expression in infected and surrounding cells, OASL connects and activates RIG-I by simulating pUb, further increasing the expression of IFN [33].

Furthermore, *BISPR* is also correlated with a *cis*-target, the upregulated neighboring gene *BST2*, sharing a bidirectional promoter. We observed that *BISPR* does not have binding potential with *BST2*, suggesting that regulation could take place at the transcriptional level. In fact, Kambara et al. (2015) [31] demonstrated that BISPR may increase expression of *BST2* by attracting transcription factors to the proximal promoter. These transcription factors may induce local chromatin remodeling or activate transcriptional complexes directly to the transcription start site.

The positive regulation of *BST2* by *BISPR* is important to control the release of CHIKV from infected to uninfected cells. BST2, a host membrane protein induced by interferon, blocks the exit of many enveloped viruses by directly tethering budded particles to the cell surface [34, 35]. Notwithstanding, Mahauad-Fernandez et al. (2014) [36] demonstrated that BST2 was co-localized with infectious CHIKV, thus suppressing CHIKV release from infected mouse embryonic fibroblasts and macrophages during acute infection. We suggest that *BISPR* regulates *BST2* expression in whole blood to prevent the release of new viruses to neighboring cells.

*BISPR*, *OASL*, and *BST2* are also upregulated during DENV and ZIKV acute infection in whole blood of pediatric patients. Like in CHIKV infection, RLRs are important receptors that recognize DENV and ZIKV RNAs, leading to activation of IRF3 and NF-kB and IFN-I production [37]. This indicates that *BISPR*/*OASL* interaction is also important to IFN-I production and regulation of antiviral immunity response in these other two arboviral infections, mediated by DENV and ZIKV. In regards to the upregulated *BST2* expression, Pan et al. (2012) [38] demonstrated the ability of BST2 to control DENV release from human hepatoma cells, and Herrlein et al. (2022) [39] showed an intense increase in BST2 mRNA expression during ZIKV infection, although it does not culminate in increased BST2 protein due to enhanced lysosomal degradation, as a viral escape strategy. Taken together, these results indicates that *BISPR*/*OASL* and *BISPR*/*BST2* co-expression is common among these arboviral infections and places *BISPR* as a candidate lncRNA in the regulation of coding transcripts involved in antiviral response.

## Conclusion

Our findings indicate that CHIKV infection alters mRNA expression, which may be regulated at transcription, or transcript availability, by differentially expressed lncRNAs. Regulation of mRNAs by lncRNAs can facilitate viral recognition through the RIG-I-like receptor, which stimulates interferon production and antiviral immune response. We were able to demonstrate *in silico* the binding potential between co-expressed lncRNAs and mRNAs participating in pathways related to antiviral immunity, pointing to the need for future studies focused on the functional validation of these interactions. Our research also raises the question of whether altered lncRNA-mRNA interactions mediate the establishment of chronic illness.

## Supporting information

S1 Table Differentially expressed coding transcripts in the contrast acute x convalescent. (XLSX)

S2 Table Differentially expressed lncRNAs in the contrast acute x convalescent. (XLSX)

S3 Table LncRNA-mRNA correlation between all lncRNAs and mRNAs differentially expressed. (XLSX)

S4 Table LncRNA-mRNA *cis* interactions in a 100kbps up and downstream window. (XLSX)

S5 Table LncRNA-mRNA *trans* interactions with a potential of ligation. (XLSX)

S6 Table REACTOME and GO terms enriched by all mRNAs targeted by lncRNAs in *cis* or *trans*. (XLSX)

S7 Table KEGG pathways enriched by all mRNAs targeted by lncRNAs in *cis* or *trans*. (XLSX)

S8 Table Interferon stimulated genes according to the Interferome database. (XLSX)

S9 Table Differentially expressed genes analyzed in GEO2R in the contrasts: DENV Acute x DENV convalescent and ZIKV acute x ZIKV convalescent. (XLSX)
